# Smoking and Health-Related Quality of Life in the General Population. Independent Relationships and Large Differences According to Patterns and Quantity of Smoking and to Gender

**DOI:** 10.1371/journal.pone.0091562

**Published:** 2014-03-17

**Authors:** Joël Coste, Laurent Quinquis, Samuel D'Almeida, Etienne Audureau

**Affiliations:** 1 Biostatistics and Epidemiology Unit, Assistance Publique-Hôpitaux de Paris, Hôtel Dieu, Paris, France; 2 Research unit APEMAC, EA 4360, Université Paris-Descartes, Sorbonne Paris Cité, Lorraine Université, Paris, France; Clinica Universidad de Navarra, Spain

## Abstract

**Background:**

Relationships between smoking and health-related quality of life (HRQoL) in the general population remain unclear.

**Objectives:**

To quantify the independent associations between smoking patterns and HRQoL and to identify any threshold or non-linear tendencies in these associations.

**Methods:**

A national representative, cross-sectional household survey of the French general non institutionalized population included 7525 men and 8486 women, aged 25–64 year in 2003. Scores on the eight subscales of the Medical Outcomes Study 36-item Short Form were the primary outcomes. Linear regression analyses were used to evaluate the associations between HRQoL and smoking history, quantity of smoking and smoking cessation while controlling for various socio-economic variables, depression, alcohol dependence and pathological conditions. Analyses were conducted in 2013.

**Results:**

Independent associations between smoking and HRQoL were found, including small positive associations for occasional or light smoking (up to 5 cigarettes per day), and larger and diffuse negative associations above this threshold. Much weaker associations and higher thresholds for negative HRQoL were found for women than for men. For ex-smokers of both genders, HRQoL was found to be better between 2 and 5 years after quitting.

**Conclusions:**

Smoking was independently related to HRQoL, with large differences according to the pattern and quantity of smoking, and to gender. These results may have considerable relevance both for public health action and care of smokers.

## Introduction

Despite substantial declines in the prevalence of smoking in several countries, tobacco smoking continues, worldwide, to be the most harmful health behavior associated with premature disease and death [1], [2]. Relationships between smoking and health-related quality of life (HRQoL) have only recently been investigated in general populations [3]–[16]. However, most studies appear not to have taken into account co-intoxication (especially alcohol) and the burden of morbidities often associated with and/or partially caused by smoking. Depression, in particular, which has complex links with smoking [17], has never been considered as a potential confounder. Hence, the independent relationship between smoking or its cessation and HRQoL has still not been accurately estimated. In addition, the shape of the relationship between smoking and HRQoL has not been thoroughly investigated, preventing conclusions regarding possible thresholds or non-linear tendencies for these relationships. Consequently, the association between smoking and HRQoL still needs to be modeled more accurately. An impaired HRQOL associated with smoking could be an additional rationale for encouraging smokers to quit or reduce smoking. A better HRQoL in ex-smokers might also encourage quitting smoking.

The primary aim of our study was thus to investigate thoroughly these relationships in the French general population aged 25–64 years, an age range for which selection or survival biases due to smoking should be minimal. The specific aims were 1) to disentangle the *independent relationship* of smoking with HRQoL from the confounding or mediating role of depression, alcohol co-intoxication and chronic medical conditions; 2) to identify any threshold or non-linear shape in this relationship, and 3) to investigate HRQoL of ex-smokers and its links with quantity of lifetime smoking and time since quitting. We used a large representative sample of the French population for which a broad variety of socio-economic and health status variables was recorded, and for which HRQoL, evaluated by the Medical Outcomes Study Short Form 36 (SF-36), was available.

## Methods

### Setting and Participants

**Figure 1:**
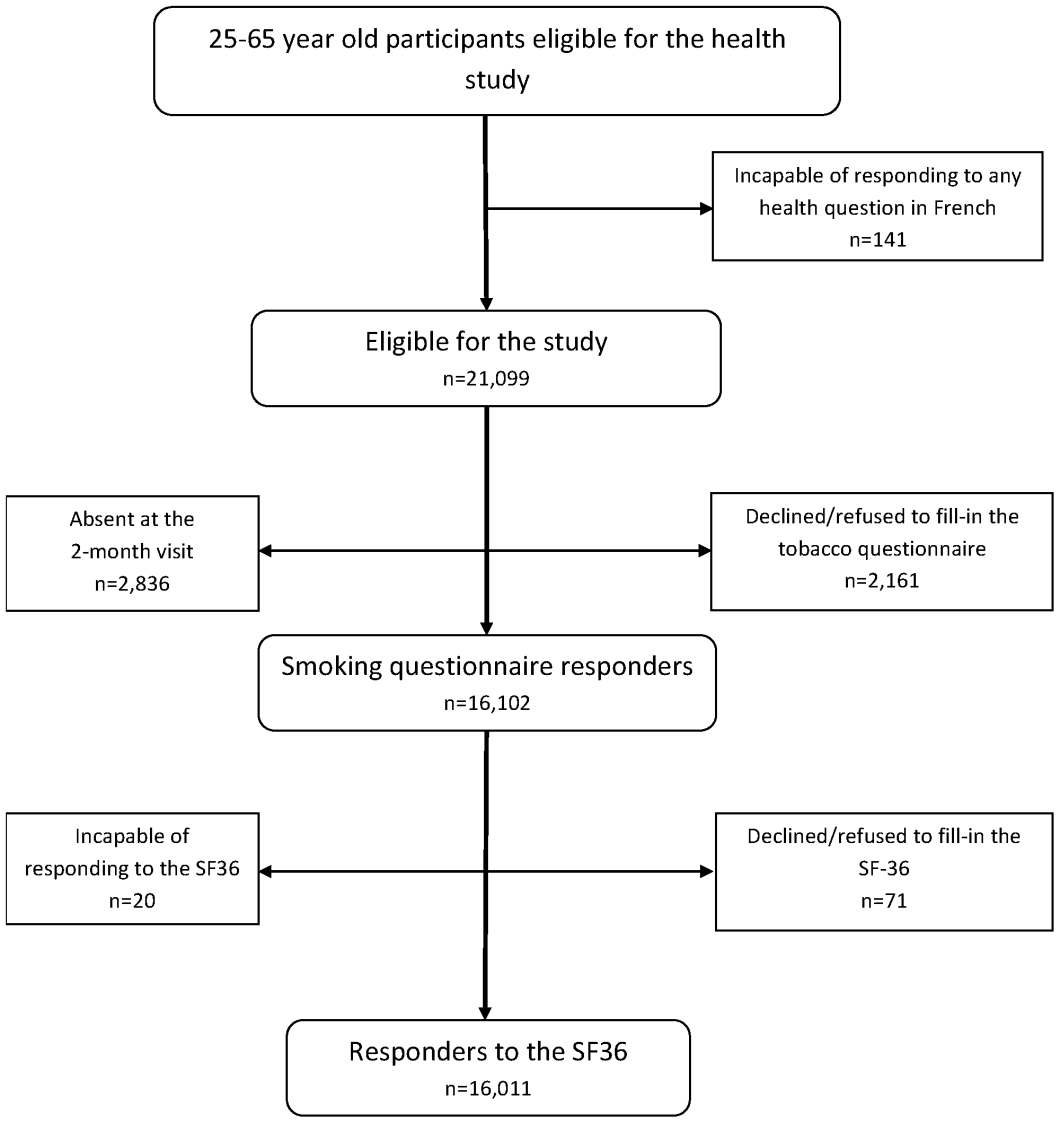
Flowchart of the study design.

The Decennial Health Survey is a national survey of households, representative of the French population, performed by the French National Institute of Statistics and Economic Studies (INSEE) on a 10-year basis since 1970 [18]. The sampling design is clustered and stratified (on region and size of urban unit) and random, all individuals in the households selected being included in the survey. During a first home visit, specifically trained interviewers collected socio-demographic, economic, health behavior and medical history data from all eligible subjects; all subjects were also given several self-administered questionnaires which were collected during a subsequent home visit 2 months later (Figure 1). The protocol respected the principles of the Declaration of Helsinki, and participants gave their informed consent.

### Ethics statement

This study was performed by the National Institute for Statistics and Economic Studies. All of the participants gave written informed consent before data collection. The data were made available in the public research domain without any identification of personal information.

### Measurements and data collected

The interviewers recorded socio-demographic characteristics (household income, education, marital status), smoking status, alcohol dependence according to the CAGE score [19], and present and past medical conditions, which were coded using the ICD-10 (international classification of diseases, 10th revision) and used to compute the Elixhauser comorbidity score [unweighted count of present chronic conditions (0 to 30)] [20]. Smokers and ex-smokers were given a questionnaire to obtain detailed information about smoking history. Present and past average numbers of cigarettes, cigars, or pipes of tobacco smoked per day and duration of smoking was recorded for current and former smokers. Time since quitting was also recorded for ex-smokers. Participants were further classified into never-smokers, ex- (former) smokers, current daily smokers (at least one cigarette [or cigar/pipe] smoked daily), current occasional (non-daily) smokers and occasional ex-daily smokers. Pack-years (or cigar/pipe equivalents, 1 cigar = 1 pipe = 3 cigarettes) were used as a measure of lifetime smoking.

Other self-administered questionnaires used included the Center for Epidemiologic Studies Depression scale (CES-D) [21] and the Medical Outcomes Study (MOS) 36-item short-form (SF-36) questionnaire [22], [23]. The SF-36 is the most widely used generic health status measure, and one of its strengths is that it covers a broad array of health dimensions, some of which may be affected by smoking. It consists of 35 items divided into eight dimensions: physical functioning, role limitations relating to physical health, bodily pain, general health perceptions, vitality, social functioning, role limitation relating to mental health, and mental health. The remaining item assesses health transition. Each question is rated on an ordinal scale with 2 to 6 categories; the score of each dimension is the sum of the item scores of the related dimension further normalized to a score of 0 to 100, with higher values representing better perceived HRQoL. As recommended, the score of each dimension was computed if at least half of the items of the related dimension were available [24]. Physical and mental component summary scores, which lack validity in France [23], were not calculated.

### Statistical Analysis

To help interpretation, the scores of the SF-36 dimensions are presented as standard deviation scores (SDS), calculated by dividing the difference between the subject's score and the mean score of the general population of the same sex and age group by the standard deviation of the general population group. French general population reference values for age (10-year interval groups) and sex were used [23]. The standard cut-off score of 16 for the CES-D was used to indicate a depressive condition.

The general strategy for disentangling the *independent relationship* of smoking with HRQoL from the confounding of socio-demographic and economic background and also from the confounding or mediating role of depression, co-intoxication and chronic conditions, consisted in the construction of regression models including variables following the chrono-logical order of the phenomena/events. Sequential linear regression models were therefore constructed in several steps. Firstly, models were constructed including only the smoking variable and socio-demographic and economic background variables that may confound the relationship (age, education, matrimonial status, occupational category, employment, income). Secondly, depressive condition variables were also introduced in addition to predictors identified as significant by the first stage and the interaction with smoking tested. Thirdly, body mass index (BMI) and CAGE score were added to predictors found by the second stage to be significant: these health behaviors were tested for their relations with smoking, and interactions between these variables and smoking were also tested. Fourthly, chronic conditions, which may explain part of the relationship between smoking and HRQoL (mediation effect), were added to predictors identified as significant by the third stage, either as a quantitative variable (Elixhauser score) or as a group of common conditions which were tested using a backward procedure. Finally, interactions between smoking and independent morbidities were tested. Collinearity was checked for each step, and was not found among smoking and other independent variables. To model accurately the relationships between the quantity of smoking and HRQoL in current daily smokers, piecewise linear regressions (according to Nakamura [25]) were used to test for potential thresholds (i.e., breaks in a linear regression) adjusting for all socioeconomic variables, CESD, BMI, co-intoxication and comorbidities independently associated with HRQoL identified as previously described. The relationship between HRQoL for ex-smokers and both of quantity of lifetime smoking and time since quitting was estimated using the same procedure as for the quantity of smoking in current smokers. Some of the scores of SF-36 subscales were skewed, and residual plots from the corresponding regression analyses were systematically checked for departure from normality (in most cases, there was no departure of residuals from normality and regression diagnostics were met). Analysis of non and partial responses to the HRQoL questionnaire (missing items, missing questionnaires, inconsistent responses) reported previously [26] indicated that the biases associated with each pattern of missing responses ran in opposite directions and partially neutralized each other; this explains why sensitivity analysis involving the use of multiple imputation for missing HRQoL data [27] provided similar results to those obtained without imputation (and is therefore not presented here). Also, the clustering effect of household upon estimates of predictors was found to be negligible in multilevel modeling; only results from standard fixed effects models are reported here.

The analysis was restricted to subjects aged 25–64 years. Older subjects were excluded to limit the survival biases due to smoking and to limit the potential confounding effect of (unassessed) chronic conditions associated with smoking; younger subjects were excluded to allow better control of socio-economic confounding, for example due to level of education and occupation.

Due to large differences in the relationships between smoking and HRQoL according to gender and age, all analyses were stratified by gender and age group (25–44 and 45–64 yrs). STATA (Version 8.0, Stata Corporation, College Station, TX) and SAS statistical software (Version 9.2 SAS Institute Inc., Cary, NC) were used. Analyses were conducted in 2013.

## Results

**Figure 2:**
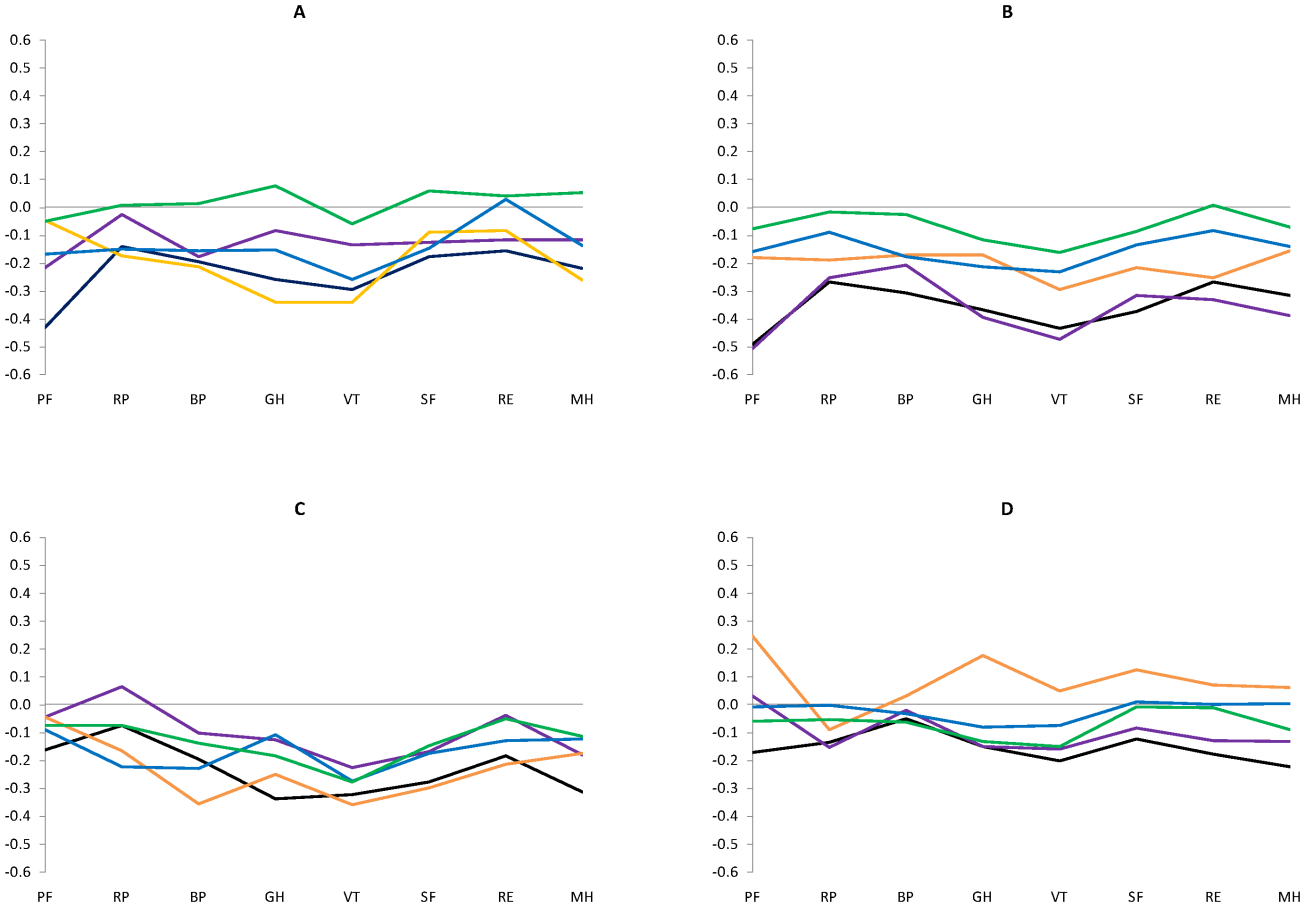
Standardized SF-36 scale scores according to smoking status: current daily smokers (black), occasional smokers (purple), occasional ex-daily smokers (orange), ex-smokers (blue), never smokers (green). Panel A, men 25–44 yrs; Panel B, men 45–64 yrs; Panel C, women 25–44 yrs; Panel D, women 45–64 yrs. PF physical functioning; RP role limitations due to physical problems; BP bodily pain; GH general health; VT vitality; SF social functioning; RE role limitations due to emotional problems; MH mental health.

Figure 1 shows a flowchart summarizing the study. Among 21,240 eligible participants, 141 were incapable of responding to any health-related question in French, 4,997 did not respond to the smoking questionnaire (most because they were absent at the 2-month visit) and another 91 did not respond to the SF-36, leaving 16,011 subjects (75%) to be included in the study. Demographic and socio-economic characteristics, alcohol status, and frequently reported morbidities are presented according to smoking status in Table 1. Most females were never smokers, whereas males were often daily and ex-smokers. Subjects of younger age, less educated, blue collar and unemployed with lower income and living alone at the time of the survey were more often daily smokers, as were those with alcohol dependence, depression and chronic pulmonary disorders. By contrast, those with cancer, cardiac diseases and diabetes were more often either never smokers and (mostly) ex-smokers. Figure 2 presents the SF-36 scale (unadjusted) scores according to gender and age group (25–44 and 45–64 yrs). Whereas daily smokers, in general, had lower scores than other groups, there was considerable diversity regarding other patterns of smoking in both gender and age groups. In particular, male never smokers had consistently higher HRQoL scores than smokers, whereas HRQoL scores were often lower for female never smokers than occasional smokers.

**Table 1:** Characteristics of 25–65 year-old subjects included in the study (n = 16,011), according to smoking status.

	Daily smoker (n = 4,089)	Occasional smoker (n = 686)	Occasional ex-daily (n = 272)	Ex-smoker (n = 2750)	Never smoker (n = 8214)
**Gender**					
Man	2248 (29.9)	335 (4.5)	137 (1.8)	1697 (22.6)	3108 (41.3)
Woman	1841 (21.7)	351 (4.1)	135 (1.6)	1053 (12.4)	5106 (60.2)
**Age**					
25–44	2635 (31.1)	426 (5.0)	173 (2.0)	1206 (14.2)	4044 (47.7)
45–64	1454 (19.3)	260 (3.5)	99 (1.3)	1544 (20.5)	4170 (55.4)
**Education**					
No diploma	410 (33.7)	69 (5.7)	19 (1.6)	162 (13.3)	558 (45.8)
Primary school	472 (21.1)	83 (3.7)	21 (0.9)	355 15.9()	1302 (58.3)
Lower secondary level	347 (29.4)	50 (4.2)	22 (1.9)	196 (16.6)	565 (47.9)
Intermediate secondary level	1563 (29.5)	201 (3.8)	98 (1.9)	970 (18.3)	2461 (46.5)
Upper secondary level	400 (26.9)	62 (4.2)	22 (1.5)	258 (17.4)	745 (50.1)
Lower tertiary level	669 (20.1)	162 (4.9)	71 (2.1)	577 (17.4)	1844 (55.5)
Upper tertiary level	228 (17.9)	59 (4.6)	19 (1.5)	232 (18.2)	739 (57.9)
**Occupation**					
Farmers	59 (16.0)	17 (4.6)	6 (1.6)	44 (11.9)	243 (65.9)
Craftsmen, shopkeepers	184 (27.4)	29 (4.3)	12 (1.8)	120 (17.9)	326 (48.6)
Senior managers, professionals	406 (18.5)	119 (5.4)	36 (1.6)	439 (20.0)	1197 (54.5)
Middle managers, professionals	764 (23.7)	129 (4.0)	62 (1.9)	615 (19.1)	1652 (51.3)
White-collar	966 (25.7)	151 (4.0)	60 (1.6)	513 (13.6)	2069 (55.0)
Blue-collar	949 (35.7)	121 (4.6)	52 (2.0)	456 (17.2)	1077 (40.6)
Unemployed	404 (36.6)	56 (5.1)	18 (1.6)	151 (13.7)	474 (43.0)
Inactive due to sickness	110 (31.3)	22 (6.3)	7 (2.0)	59 (16.8)	153 (43.6)
Other inactives, housewives, students	247 (15.6)	42 (2.7)	19 (1.2)	353 (16.0)	1023 (64.5)
**Marital status**					
Married or living in couple	3074 (24.0)	522 (4.1)	218 (1.7)	2383 (18.6)	6625 (51.6)
Single, divorced, separated, widowed	999 (31.8)	164 (5.1)	54 (1.7)	367 (11.5)	1589 (49.9)
**Income (per household unit per year, €)**					
<10,000	1097 (31.7)	170 (4.9)	36 (1.0)	463 (13.4)	1695 (49.0)
[10,000–15,000[	1205 (27.7)	167 (3.8)	88 (2.0)	748 (17.2)	2149 (49.3)
[15,000–20,000[	795 (23.6)	134 (4.0)	60 (1.8)	611 (18.2)	1762 (52.4)
≥20,000	992 (20.5)	215 (4.5)	88 (1.8)	928 (19.2)	2608 (54.0)
**Body mass index**					
<18.5	182 (36.3)	34 (6.8)	11 (2.2)	51 (10.2)	224 (44.6)
[18.5–25[	2512 (28.1)	399 (4.5)	153 (1.7)	1273 (14.2)	4609 (51.5)
[25–30[	1047 (21.6)	193 (13.0)	81 (1.7)	1061 (21.9)	2462 (50.8)
[30–35[	282 (20.8)	49 (3.6)	19 (1.4)	287 (21.1)	721 (53.1)
≥35	63 (17.8)	11 (3.1)	8 (2.3)	77 (21.8)	194 (55.0)
**Alcohol dependence (CAGE Score ≥2)**					
Yes	529 (42.3)	63 (5.0)	32 (2.6)	307 (24.6)	319 (25.5)
No	2918 (25.0)	514 (4.4)	216 (1.8)	2116 (18.1)	5916 (50.7)
**Cancer (solid tumors)**					
Yes	30 (15.5)	8 (4.1)	4 (2.1)	49 (25.3)	103 (53.1)
No	4059 (25.7)	678 (4.3)	268 (1.7)	2701 (17.1)	8111 (53.1)
**Diabetes**					
Yes	83 (21.7)	14 (3.7)	6 (1.6)	97 (25.3)	183 (47.8)
No	4006 (25.6)	672 (4.3)	266 (1.7)	2653 (17.0)	8031 (51.4)
**Hypertension**					
Yes	258 (17.3)	39 (2.6)	21 (1.4)	294 (19.7)	877 (58.9)
No	3831 (26.4)	647 (4.5)	251 (1.7)	2456 (16.9)	7337 (50.5)
**Congestive heart failure or arrhythmia or valve disease**					
Yes	45 (20.3)	8 (3.6)	3 (1.4)	48 (21.6)	118 (53.1)
No	4044 (25.6)	678 (4.3)	269 (1.7)	2702 (17.1)	8096 (51.3)
**Chronic pulmonary disorders**					
Yes	296 (32.1)	43 (4.7)	15 (1.6)	137 (14.9)	430 (46.7)
No	3793 (25.1)	643 (4.3)	257 (1.7)	2613 (173)	7784 (51.6)
**Depression (CES-D ≥16)**					
Yes	967 (30.3)	140 (4.4)	51 (1.6)	487 (15.3)	1547 (48.5)
No	2983 (24.4)	506 (4.1)	208 (1.7)	2184 (17.9)	6329 (51.8)
**Count of chronic conditions (Elixhauser score)**					
0	3192 (26.1)	546 (4.4)	204 (1.7)	2037 (16.6)	6272 (51.2)
1–2	869 (23.9)	134 (3.7)	66 (1.8)	692 (19.0)	1881 (51.6)
≥3	28 (23.7)	6 (5.1)	2 (1.7)	21 (17.8)	61 (51.7)

Figures are numbers (percentages of that characteristic in each smoking status group) unless stated otherwise.

Table 2 shows the multivariate associations between smoking status and HRQoL, stratified by gender and age group. The first striking result is that the number and the magnitude of the associations identified as significant were very substantially reduced by adjustments for depression (models B) and BMI, alcohol dependence and comorbidities (models C). In particular, many associations observed in the mental dimensions of SF-36 were found to be confounded by depression and also by alcohol dependence. Taking into account the possible mediating effect of comorbidities did not substantially modify the estimates of effects (data not shown). However, male daily smokers had lower physical functioning and general health scores, independently of the latter factors, albeit with a moderate effect size (−0.21 SDS). Also, after multiple adjustment (models C): 1) young male daily smokers showed slightly (+0.12 SDS), but significantly, higher HRQoL in the role limitations (physical) scale, a dimension which includes work limitations due to physical problems; 2) young male ex-smokers had (as could be suspected) lower mental HRQoL (−0.13 to −0.28 SDS for general health, vitality and mental health); and 3) occasional smokers, and especially older ones, had higher mental and social HRQoL (+0.35 SDS). A striking result is the major differences between men and women regarding the associations between smoking and HRQoL: these associations appeared to be much weaker and, after adjustment for depression and alcohol dependence, even insignificant in women. Of particular interest is the absence of association between smoking and physical dimensions of HRQoL in women. Also of note is the absence of any significant interaction of depression and alcohol dependence with smoking status on HRQoL scores.

**Table 2:** Multivariate associations between smoking status and HRQoL (SF-36 scales), stratified by gender and age group.

1. Men		25–44 yrs				45–64 yrs			
Subscale	*Model*	Daily smoker	Occasional smoker	Occasional ex-daily	Ex-smoker	Daily smoker	Occasional smoker	Occasional ex-daily	Ex-smoker
**Physical functioning**	***A***	−0.25(−0.36 to −0.13)				−0.26(−0.37 to −0.15)			
	***B***	−0.23(−0.35 to −0.11)				−0.20(−0.30 to −0.09)			
	***C***	−0.21(−0.37 to −0.04)				−0.18(−0.35 to −0.01)			
**Role limitations – physical**	***A***				−0.14(−0.24 to −0.03)	−0.14(−0.24 to −0.03)			
	***B***								
	***C***	0.12(0.01 to 0.23)							
**Bodily pain**	***A***	−0.13(−0.21 to −0.05)			−0.13(−0.23 to −0.03)	−0.18(−0.27 to −0.08)			−0.15(−0.24 to −0.06)
	***B***	−0.09(−0.17 to −0.01)				−0.13(−0.22 to −0.03)			
	***C***						0.35(0.01 to 0.70)		
**General health**	***A***	−0.26(−0.34 to −0.19)		−0.40(−0.64 to −0.15)	−0.24(−0.34 to −0.14)	−0.16(−0.25 to −0.07)			−0.09(−0.17 to −0.00)
	***B***	−0.22(−0.29 to −0.14)		−0.34(−0.57 to −0.10)	−0.22(−0.31 to −0.12)	−0.11(−0.20 to −0.03)			−0.08(−0.16 to −0.00)
	***C***	−0.21(−0.32 to −0.11)			−0.28(−0.42 to −0.13)				
**Social functioning**	***A***	−0.18(−0.26 to −0.11)	−0.17(−0.32 to −0.01)		−0.20(−0.30 to −0.11)	−0.15(−0.25 to −0.05)			
	***B***	−0.11(−0.18 to −0.05)			−0.16(−0.25 to −0.07)				
	***C***						0.37(0.04 to 0.70)		
**Vitality**	***A***	−0.21(−0.28 to −0.13)		−0.28(−0.52 to −0.05)	−0.24(−0.33 to −0.05)	−0.13(−0.23 to −0.04)			
	***B***	−0.15(−0.22 to −0.08)		−0.22(−0.44 to −0.01)	−0.21(−0.29 to −0.12)				
	***C***				−0.19(−0.32 to −0.05)				
**Role limitations – emotional**	***A***	−0.14(−0.22 to −0.06)				−0.18(−0.28 to −0.08)			
	***B***					−0.11(−0.20 to −0.02)			
	***C***								
**Mental health**	***A***	−0.20(−0.27 to −0.13)	−0.15(−0.30 to −0.01)	−0.28(−0.51 to −0.05)	−0.20(−0.30 to −0.11)	−0.14(−0.24 to −0.05)			
	***B***	−0.14(−0.20 to −0.07)			−0.17(−0.25 to −0.09)				
	***C***				−0.13(−0.25 to −0.01)				

2. Women		25–44 yrs				45–64 yrs			
Subscale	*Model*	Daily smoker	Occasional smoker	Occasional ex-daily	Ex-smoker	Daily smoker	Occasional smoker	Occasional ex-daily	Ex-smoker
**Physical functioning**	***A***					−0.10(−0.20 to −0.01)			
	***B***								
	***C***								
**Role limitations – physical**	***A***				−0.17(−0.27 to −0.07)				
	***B***								
	***C***								
**Bodily pain**	***A***								
	***B***								
	***C***								
**General health**	***A***	−0.11(−0.19 to −0.04)						0.33(0.00 to 0.66)	
	***B***	−0.08(−0.15 to −0.01)						0.31(0.00 to 0.61)	
	***C***				0.16(0.05 to 0.27)				
**Social functioning**	***A***	−0.09(−0.16 to −0.01)				−0.09(−0.18 to −0.00)			
	***B***								
	***C***								
**Vitality**	***A***								
	***B***								
	***C***								
**Role limitations – emotional**	***A***	−0.10(−0.18 to −0.02)				−0.12(−0.22 to −0.03)			
	***B***								
	***C***								
**Mental health**	***A***	−0.14(−0.21 to −0.06)				−0.11(−0.21 to −0.01)			
	***B***	−0.06(−014 to 0.01)							
	***C***						0.26(0.05 to 0.47)		

The never smoker group is used as the reference.

Model A: estimates (95% confidence interval) adjusted for socioeconomic variables (education, occupation, and income); Model B: estimates (95% confidence interval) adjusted for socioeconomic variables and CESD score; Model C: estimates (95% confidence interval) adjusted for socioeconomic variables, CESD, BMI, co-intoxication and comorbidities. For the sake of readability, only significant associations are reported in the table.

**Figure 3:**
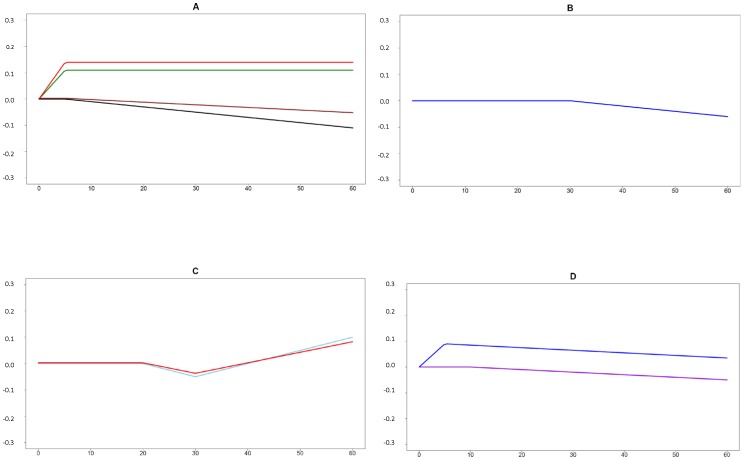
Relationships between the quantity of smoking (x-axis) and HRQoL (y-axis, standardized scores) in current daily smokers. Panel A, men 25–44 yrs; Panel B, men 45–64 yrs; Panel C, women 25–44 yrs; Panel D, women 45–64 yrs. Colors: physical functioning (black); role limitations due to physical problems (skyblue); bodily pain (green); general health (brown); vitality (violet); role limitations due to emotional problems (red); mental health (dark blue); only subscales with significant thresholds are shown.

Table 3 and Figure 3 give the main results of the analysis of thresholds in the relationships between the quantity of smoking and HRQoL for current daily smokers. Once again, few relationships remained after adjustment for depression and alcohol dependence, and the relationships were different for men and women. A threshold of 5 cigarettes per day was found for decreased HRQoL in younger male smokers in two emblematic dimensions of the SF-36: general health and physical functioning. In this group, the bodily pain scale and in the role limitations (emotional) scale (a dimension which included work limitations due to emotional problems), both increased with smoking up to 5 cigarettes per day and plateaued thereafter. (In older male smokers, only a threshold of 30 cigarettes per day was found for decreased mental health). For women, the relationships were fewer, and included: for younger women only role limitations (physical and emotional), with a first threshold of 20 cigarettes per day for decreased HRQoL and a second at 30 cigarettes per day for increased, or more probably plateaued, HRQoL (the sample size for more than 30 cigarettes per day was limited); and for older women, there was a threshold of 10 cigarettes per day for vitality, and mental health HRQoL increased up to 5 cigarettes per day, and decreased thereafter.

**Table 3:** Overview of the analysis of thresholds in the relationships between the quantity of smoking and HRQoL in current daily smokers.

	Physical functioning	Role limitations – physical	Bodily pain	General health	Social functioning	Vitality	Role limitations – emotional	Mental health
Men, 25–44 yrs	5 cig./day (−0.02)**	-	1 cig./day (0.22) +5 cig./day (−0.22) ***	5 cig./day (−0.01)	-	-	1 cig./day (0.28) +5 cig./day (−0.28)	-
Men, 45–64 yrs	-	-	-	-	-	-		30 cig./day (−0.02)
Women, 25–44 yrs	-	20 cig./day (−0.05) +30 cig./day (0.10)	-	-	-	-	20 cig./day (−0.04) +30 cig./day (0.08)	-
Women, 45–64 yrs	-	-	-	-	-	10 cig./day (−0.01)	-	1 cig./day (0.18) +5 cig./day (−0.19)

Thresholds and estimates of effects*.

* Estimates adjusted for socioeconomic variables, CESD, BMI, co-intoxication and comorbidities.

** Threshold (estimate of effect) is shown; here for example, a man aged 25–44 loses 0.02SDS of physical functioning per cigarette smoked above 5 cig./day.

*** There are two thresholds, with a plateau effect at the second threshold; here for example a man aged 25–44 gains 0.22 SDS for the bodily pain score per cigarette smoked up to 5 per day, *plus* loses 0.22 SDS per cigarette smoked above 5 cig./day.

Table 4 presents the associations between HRQoL in ex-smokers and both quantity of lifetime smoking (pack-years) and time since quitting. Whereas the quantity of lifetime smoking was almost negligibly associated with HRQoL after adjustment for depression and comorbidities, time since quitting seemed more consistently associated with (better) HRQoL, 2–5 years being the interval of time more consistently associated with better HRQoL.

**Table 4:** Associations of between HRQoL and both quantity of lifetime smoking (pack-years) and time since quitting in ex-smokers.

		Physical functioning	Role limitations – physical	Bodily pain	General health	Social functioning	Vitality	Role limitations – emotional	Mental health
**Men, 25–44 yrs**	Lifetime smoking								
	Time since quitting					1 year (ref)			1 year (ref)
						*2–5 year (0.29)*			*2–5 year (0.26)*
						*6–10 year (0.25)*			
**Men, 45–64 yrs**	Lifetime smoking	1–5 pack-year (ref)							
		*5–9 pack-years (0.28)* **							
	Time since quitting								
**Women, 25–44 yrs**	Lifetime smoking			1–5 pack-year (ref)					
				*10–19 pack-years (−0.31)*					
	Time since quitting	1 year (ref)		1 year (ref)					
		*2–5 year (0.41)* ***		*2–5 year (0.27)*					
**Women, 45–64 yrs**	Lifetime smoking								
	Time since quitting						1 year (ref)		1 year (ref)
							*>10 years (0.26)*		*>10 years (0.27)*

Categories of these variables independently associated with HRQoL and estimates of effects*.

* Categories independently associated with HRQoL (estimate of effect) are shown. Estimates are adjusted for socioeconomic variables, CESD, BMI, co-intoxication and comorbidities (full adjustment).

** The physical functioning score for a man aged 45–64 increases by 0.28 SDS after quitting if he previously smoked between 5–9 pack-years (1–5 pack-years being the reference category).

*** The physical functioning score for a woman aged 25–44 increases by 0.41 SDS between 2 and 5 years after quitting smoking (1 year being the reference category).

## Discussion

In this large, nationally representative sample of the French general population aged 25–64 years, careful control of confounding factors, especially socio-economic status, depression and alcohol dependence and rigorous accounting for possible mediating effects of morbidities allowed *independent associations* between smoking and HRQoL to be dissected out. Such associations appeared probably in their purest forms in the 25–44 year-old group, in which morbidities are (still) minimal. For men, we report that physical functioning and general health was about 0.2 SD lower, and physical role functioning very slightly higher in daily smokers than never smokers, and that general health and mental dimensions of HRQoL were 0.15–0.3 SD lower in ex-smokers than never smokers. The association for ex-smokers appeared to be time-dependent, the positive effects of smoking cessation being seemingly perceived after two years and especially between 2 and 5 years after quitting. For male daily smokers, a linear decrease of HRQoL above the threshold of 5 cigarettes per day was consistently found for important dimensions of the SF-36. On the other hand, weak positive independent relationships were found for 25–44 year-old male daily smokers for the bodily pain scale and emotional role functioning scales and for 45–64 year-old occasional smokers of both genders despite their appearing less consistent through all the HRQoL dimensions.

**Figure 4:**
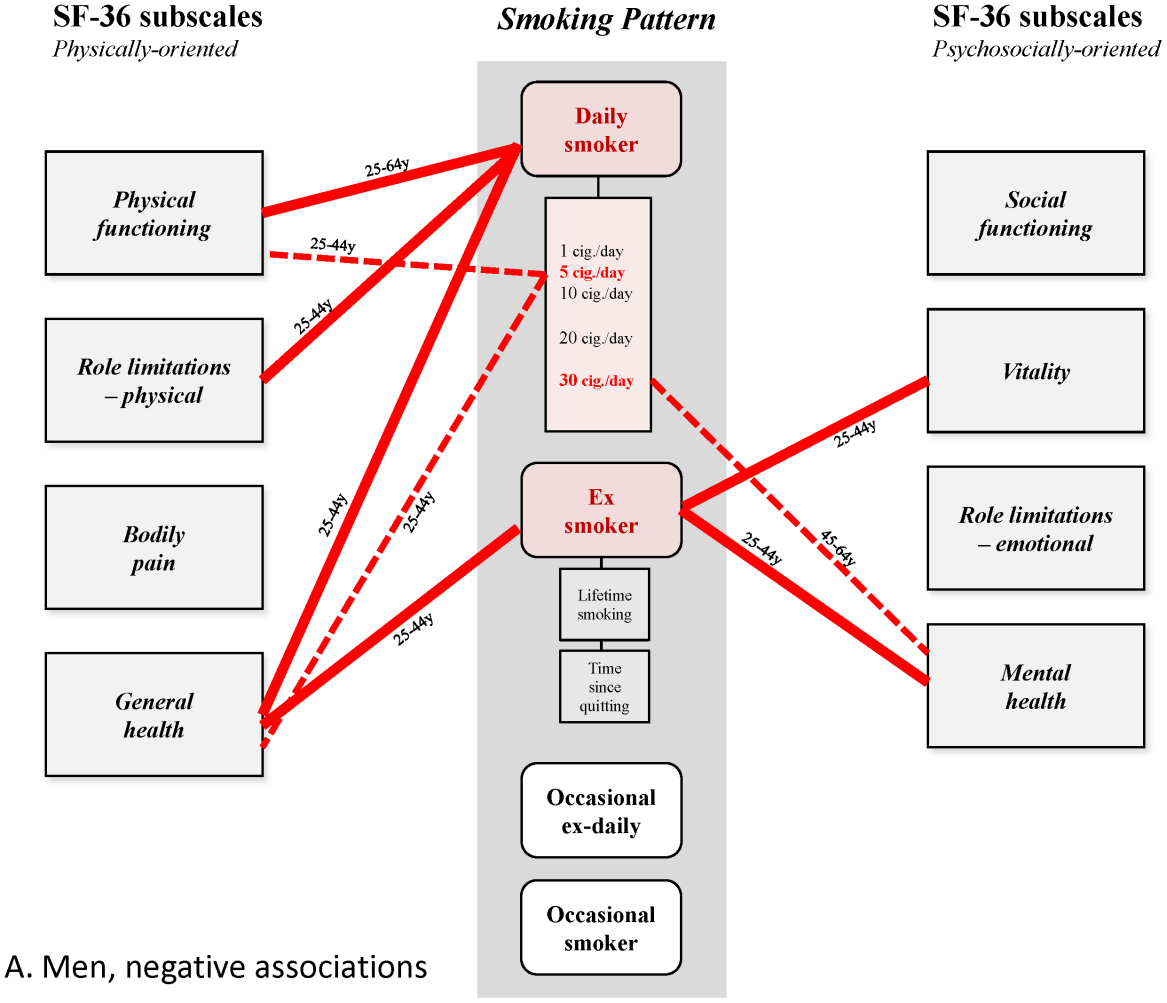
Patterns of relationships found in this study. Men, positive associations.

**Figure 5:**
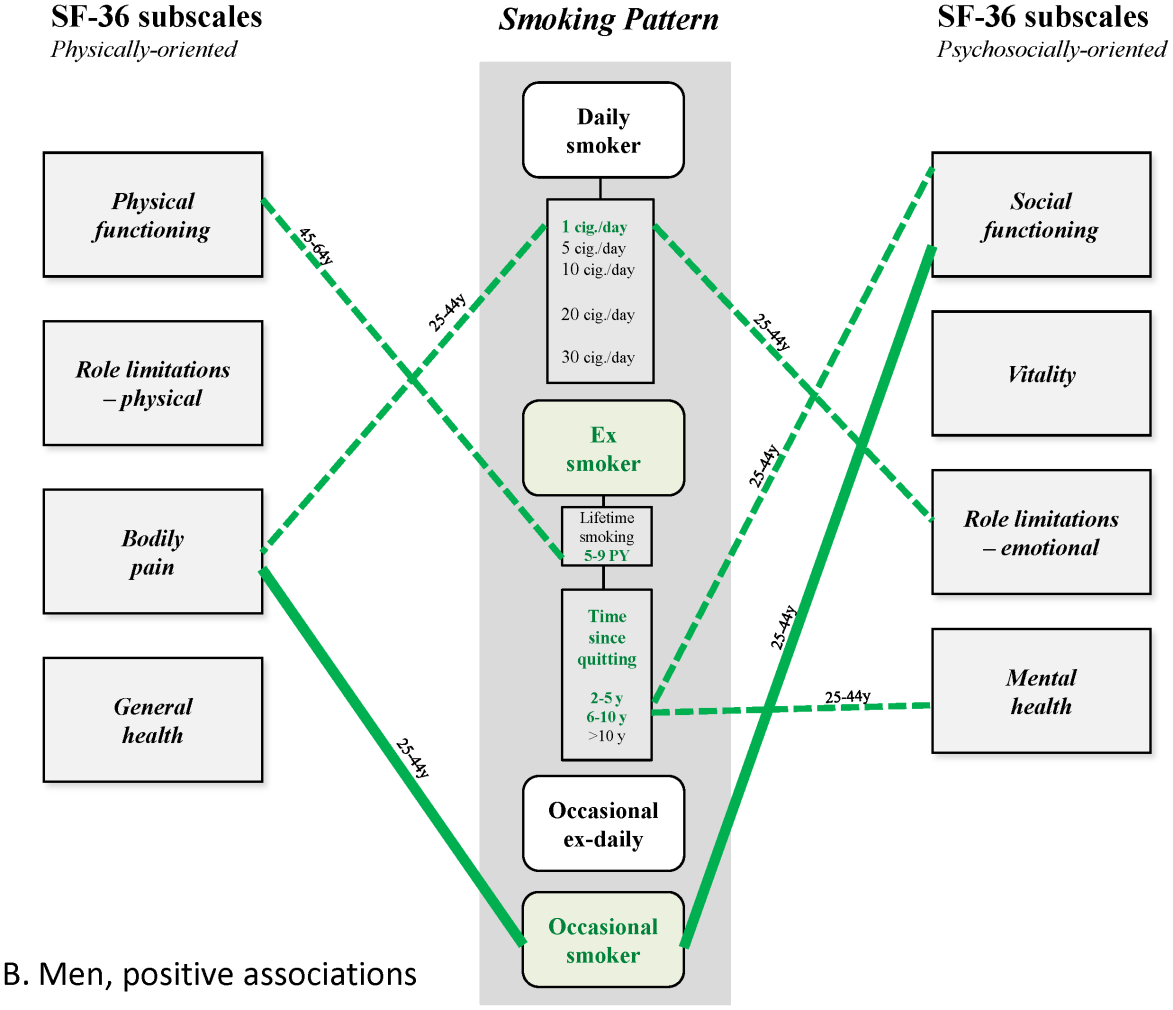
Patterns of relationships found in this study. Men, negative associations.

**Figure 6:**
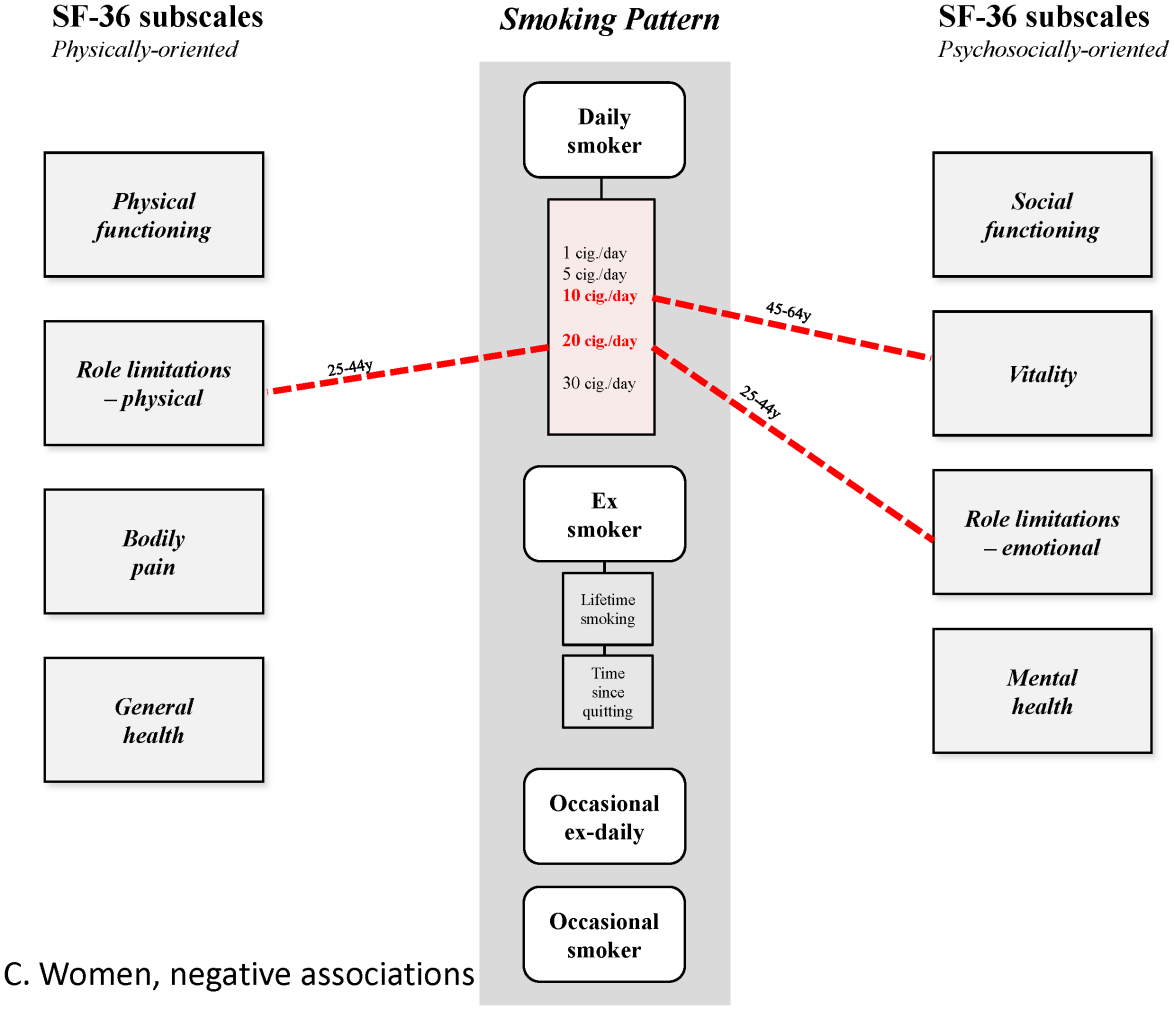
Patterns of relationships found in this study. Women, positive associations.

**Figure 7:**
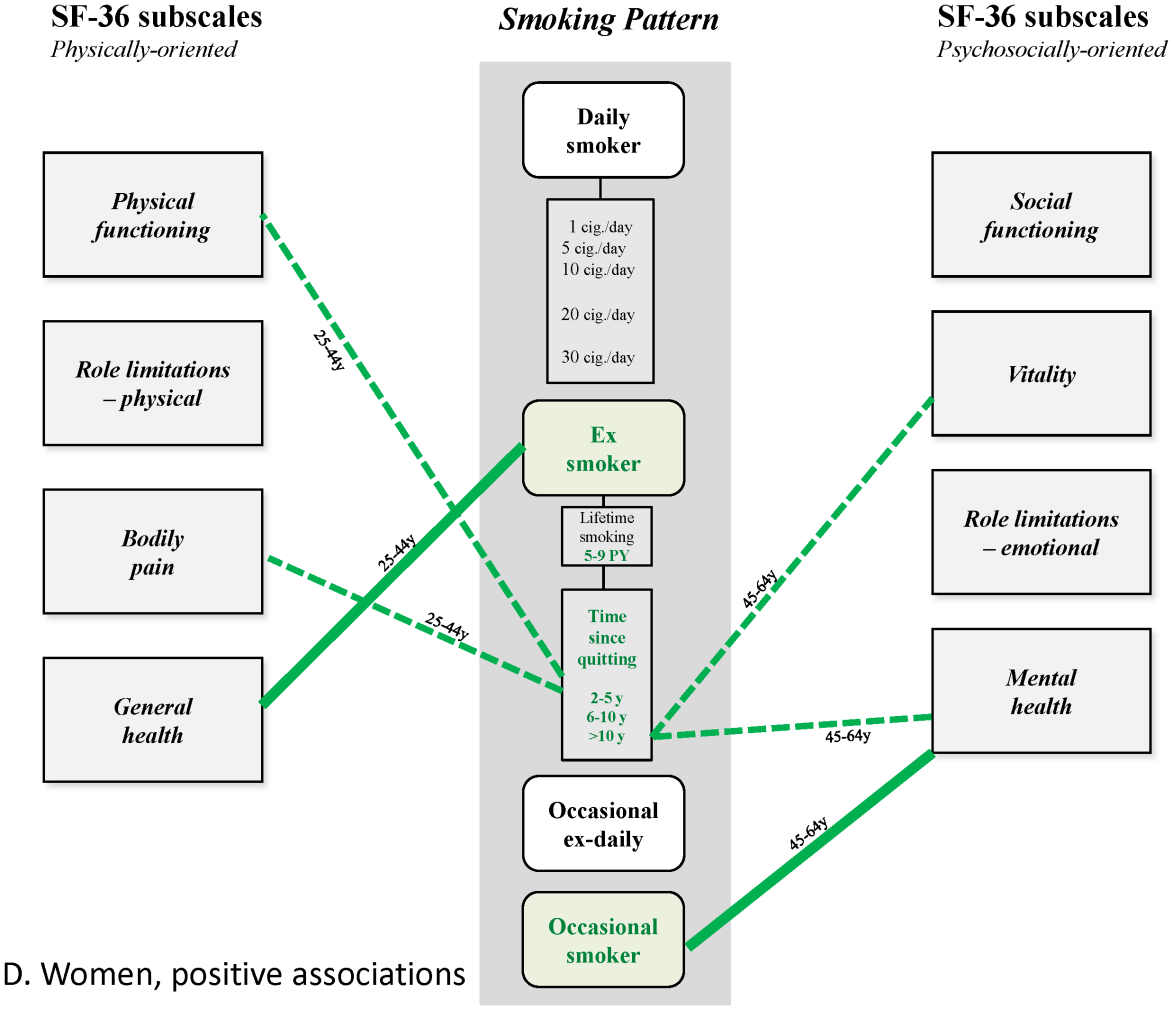
Patterns of relationships found in this study. Women, negative associations.

These results thus reveal a differentiated pattern of (small) positive and (larger) negative associations that has not been previously described (Figures 4–7). This is undoubtedly because earlier studies did not disentangle the small positive associations from much larger ones, due to confounding of alcohol dependence and depression, or effects possibly mediated by induced morbidities, and often did not analyze associations separately for age group and gender. Epidemiological studies have shown the high frequency of the co-occurrence of alcohol use and smoking in the general population [28], [29], and despite conflicting explanations advanced for this phenomenon [30], it should be taken into account, and controlled for when analyzing effects of smoking. The strong association between smoking and depression is also well-established. It is partly explained by common factors [31], [32] and is probably bidirectional [17], with a stronger effect being depression predicting smoking initiation [33], progression to daily smoking [30] and continuation [34]. This association deserves careful attention due to the massive, well-established impact of depression on HRQoL [35], [36]. The strategy underlying our analysis involved the construction of successive nested multivariate models of HRQoL to quantify the associations, independent or not, between HRQoL and all of smoking, depression and alcohol dependence in the general population. This strategy also allowed, in theory at least, precise estimation of the influence of morbidities related to smoking. However, these morbidities appeared to have only modest or negligible influence of on the observed associations; this may have been due to their relatively low frequency and low level of severity in the studied sample. Indeed, this result may not be general, and not apply, for example, to older populations, or populations where smoking is more prevalent.

Except Heikkinen et al. [9] and recently Gan et al. [16], no previous authors thoroughly examined the relationships between smoking and HRQoL according to gender. Similarly to Heikkinen et al. [9] we found weaker relationships for women than for men. In particular, no association was found between smoking and physical dimensions of HRQoL for women. Also, the threshold for negative perceived HRQoL was found to be much higher for women, at 20 cigarettes per day, than the 5 per day for men. These differences appear of particular relevance both for public health action targeting, and for care of, female smokers. They are not completely unexpected because there is growing evidence that the dynamics of smoking initiation, progression and cessation differ between men and women [37]–[39]. The differences observed in perceived HRQoL are of particular importance since women have been shown to be more susceptible than men to the addictive effects of smoking [39], [40] and to the development of more morbidities than men [41]. Our findings are a further argument for including gender [39], [42] and HRQoL measurement in smoking control and research agendas. Complementary to biological and behavioral indicators, HRQoL or “perceived health” may be an important predictor of outcome in smoking prevention or cessation studies, as has been shown in many other clinical settings.

Finally, this study provides insights into the pattern of light and intermittent smoking [43]. This pattern is increasing in frequency in many countries and is increasingly being scrutinized [43]–[45]. In this study, the subtype of *ever* occasional smoking, and not the subtype occasional ex-daily smoking, was associated with a better HRQoL. There are several possible explanations of this result, including that of successful self-medication of these subjects with smoking [46].

There are several implications of our results that may help optimizing initiatives to prevent smoking initiation and encourage cessation. Public education messages generally rely on information about the risks of smoking and the benefits of quitting. Indeed, the benefits on morbidity and mortality of achieving and maintaining complete abstinence have been widely demonstrated in smokers of all ages [47]. Our findings suggest that smoking cessation may also have a positive impact on HRQoL, and this information may be valuable for encouraging smokers to quit: such beneficial effects may have a global and more direct influence on several aspects of daily living. To put these findings in perspective, despite their limited magnitude at the individual level, those positive effects on HRQoL could have substantial consequences – including economic returns [15] – at the population level, because there are currently 16 million smokers in France, of whom 14 million are considered to be daily smokers. Moreover, and consistent with the self-medication theory discussed above, we found some slight but significant positive effects of smoking on HRQoL among occasional smokers, and this is consistent with there being secondary benefits at the individual level. This issue has direct implications for optimizing the design of quitting campaigns, because it suggests that isolated information focused on the risks of smoking could be improved by enlarging the scope. For example, it may be beneficial to promote positive and accessible alternative behaviors (e.g. physical activity, social and cultural participation) to compensate for whatever is lost when quitting smoking. Similarly, the observation that there are intricate relationships between depression, alcohol consumption, smoking patterns and HRQoL, implies that better outcomes could be achieved if cessation interventions provided multiple forms of support e.g. targeting multiple behaviors and/or facilitating access to mental health services.

Several limitations of this study require discussion. First, the cross-sectional design precludes the determination of causal relationships between smoking patterns and HRQoL, despite the chronology of “exposure” (smoking being usually a long-lasting behavior) and HRQoL measurement being generally unambiguous. Second, self-reported smoking habits and morbidities elicited by trained lay interviewers (not medical doctors) were used, and no access to medical records to ascertain or complement the data collected was possible. Self-reporting biases tend to give attenuated estimated associations (biased toward null) and limit the control of confounding factors. Third, although we attempted to adjust for a number of potential confounding factors, there may have been residual confounding from unmeasured or incompletely measured underlying socio-economic or behavioral factors. However, the magnitude of any such biases was probably small enough for them to be unlikely to affect the validity of the results substantially. Fourth, the validity of SF-36 to detect associations between smoking and HRQoL needs to be considered. It is conceivable that some aspects of quality of life, or other aspects such as self-esteem, social adjustment, sexuality, etc., related to smoking might not be covered by the questionnaire. However, the SF-36 has been able to detect improvement in HRQoL in a cohort of 31 subjects quitting smoking [48]. Fifth, the interpretation of the magnitude of differences in HRQoL scores remains difficult despite the availability of population reference values and computation of standard deviation scores. Both the “minimal important difference” and the “clinically important difference” have been shown to vary between populations and contexts [49]. Consequently, the standard threshold of 0.5 standard deviation as indicative of a “clinically significant difference” (and which is derived from studies conducted in the clinical setting [50]) might not be straightforwardly applicable to our study. Also, many of the differences in HRQoL observed in this study are of 0.2–0.3 SD, and such values are far from being negligible; indeed, they may be important at the level of populations, as discussed above regarding the positive effects of smoking cessation on HRQoL. Finally, several findings of this study, which was conducted in France in 2003, are not necessarily applicable in other countries where smoking control legislation is older and stronger. Indeed, it is even questionable to which extent our results can be generalized to the current French context, since several substantial changes have been made to French tobacco control legislation since the early 2000's. These include several successive increases in the tax on cigarettes, the restriction of smoking in public places, explicit health warning labels on cigarette packages and prohibition of the sale of cigarettes to children under 18 years of age. However, it is remarkable that only minor changes have been observed in the prevalence and patterns of smoking in the adult general population over this time: the repeated national Health Barometers found the prevalence of regular smokers to be stable between 2000 and 2010 (around 35% for both years), the prevalence of occasional smokers to be stable between 2005 and 2010 (around 5% for both years), and that between 2005 and 2010 there was a trend for the prevalence of daily smokers to increase slightly, although there has been a small decrease in daily consumption (26.9% vs. 28.7%; 15.4 vs. 13.9 cig./day, respectively) [51]. Given the limited magnitude of these changes, it is likely that most of the relationships identified in the present work between HRQoL and smoking patterns are valid in the current context.

In conclusion, in this large national representative study, smoking was found to be independently related to HRQoL in a relatively complex manner. Although small positive associations in men were observed in occasional or light smokers, much larger and broad-spectrum negative associations were found above the threshold of 5 cigarettes per day. Much weaker associations and higher thresholds for negative HRQoL were found in women than in men. For both male and female ex-smokers, HRQoL was noticeably better between 2 and 5 years after quitting. These results provide supplementary arguments for HRQoL being used as an additional rationale in programs that aim to encourage smokers to quit or to reduce their smoking.
